# Comparative analysis of cytological changes in the buccal mucosa among traditional cigarette and electronic cigarette users

**DOI:** 10.18332/tid/203670

**Published:** 2025-06-23

**Authors:** Magdi M. Salih, Thamer A. Tamr, Tariq E. Elmissbah, Sabah M. Hanafy, Haytham A. Dahlawi, Eman H. Khalifa

**Affiliations:** 1 Department of Clinical Laboratory Sciences, College of Applied Medical Sciences, Taif University, Taif City, Kingdom of Saudi Arabia; 2 Pathology Department, King Abdulaziz Specialist Hospital, Taif City, Kingdom of Saudi Arabia; 3 Department of Pathology, Faculty of Medicine, Zagazig University, Zagazig, Egypt; 4 Laboratory Medicine Department, Faculty of Applied Medical Sciences, University of Al Baha, Al Baha, Kingdom of Saudi Arabia

**Keywords:** smoking, electronic cigarette, cytological changes

## Abstract

**INTRODUCTION:**

The study aimed to evaluate cytological changes in the buccal mucosa among traditional cigarette smokers and electronic cigarette smokers.

**METHODS:**

A cross-sectional study was conducted with 159 participants, including 97 smokers (users of traditional cigarettes, electronic cigarettes, or both) and 62 non-smokers. Participants were recruited from the student and staff population at the College of Applied Medical Sciences, Taif University. Buccal smears were collected from the lateral buccal mucosa using a wooden spatula and stained with the Papanicolaou technique for cytological evaluation.

**RESULTS:**

Cytological analysis using Papanicolaou (Pap) staining showed negative results in 51.6% of participants, reactive changes in 29.6%, and inflammatory changes in the remainder. Reactive changes were significantly more common in smokers (46.4%) than non-smokers (3.2%) (p=0.001), with higher prevalence in traditional cigarette users (51.4%) compared to e-cigarette users (37.5%) and dual users (50.0%). These changes were most frequent in individuals who smoked for ≥5 years (71.8%) versus <5 years (33.8%) (p=0.001).

**CONCLUSIONS:**

This study demonstrates a strong association between smoking and cytomorphological changes in the buccal mucosa, with severity linked to smoking duration and intensity, particularly in traditional and dual users. The findings highlight the cytotoxic impact of smoking on oral cells and the need for targeted public health interventions.

## INTRODUCTION

The detrimental effects of tobacco use on the buccal cavity and overall health are well-documented in the literature, along with the increased risk of oral tumors and cancers. However, there is a growing discourse surrounding the increasingly popular electronic cigarettes (e-cigarettes), particularly among youths and adults^1^. E-cigarettes are often marketed as safer alternatives to traditional cigarettes, and their use among young adults is rapidly increasing worldwide^2^. These devices operate by using battery power to heat liquids, producing an aerosol that is then inhaled. The liquid typically contains nicotine, a glycerin base, and various flavorings^3^. A study conducted in Saudi Arabia found that individuals who use e-cigarettes were significantly more likely also to smoke traditional tobacco products^4^. Oral cancers, particularly those associated with tobacco smoking, have been reported to have the highest incidence among cancers in the Gulf countries^5^. Cigarette smoking poses significant health risks, but the oral health effects of electronic cigarettes remain unclear. Evidence suggests they may increase cytogenetic and cytotoxic damage in the oral mucosa of former smokers^6^.

Histopathological examination using tissue biopsy remains the gold standard for diagnosing oral lesions. However, it is invasive, costly, and not always feasible in clinical practice^7^. Buccal smear cytology offers a noninvasive, fast, and sensitive alternative, with significant specificity for detecting atypical and precancerous changes. Cigarette smoking has been linked to cytomorphological alterations in the buccal mucosa^8-10^. Cytological changes in buccal epithelial cells, such as altered nuclear morphology and nucleocytoplasmic ratios, can be identified through Pap staining. Key features include irregular nuclear membranes, enlargement, hyperchromasia, shape variations, coarse chromatin, and prominent nucleoli, all critical for early oral cancer detection. Their absence indicates negative results^11^.

This study aims to evaluate the effects of electronic cigarettes and traditional cigarette smoke on the buccal mucosa, compared to non-smokers, by assessing cytomorphological changes.

## METHODS

A cross-sectional study was conducted involving volunteers, including traditional cigarette smokers, electronic cigarette smokers, and non-smokers, drawn from the healthy student and worker populations at the College of Applied Medical Sciences, Taif University. The study period spanned from September 2023 to June 2024.

### Study population and data collection

The study comprised 159 participants, including 97 smokers categorized as users of traditional cigarettes, electronic cigarettes, or both (dual users), and 62 non-smokers. Demographic information and related data were gathered through a structured survey.

*Inclusion criteria*

Participants were required to meet the following inclusion criteria: smokers, individuals who smoked either traditional cigarettes, electronic cigarettes, or both. Participants must have had a history of smoking for at least four years and smoked a minimum of five cigarettes per day. Non-smokers were defined as individuals who had never smoked.

*Exclusion criteria*

Participants with clinically visible alterations in the oral mucosa, individuals who smoked pipes, or those who consumed tobacco in other forms were excluded. These exclusions were made to avoid variations in tobacco concentration that could differently impact oral mucosal cells and induce additional systemic effects. Furthermore, individuals with oral lesions were not included in the study.

### Sample collection and staining procedure

**Figure 1:**
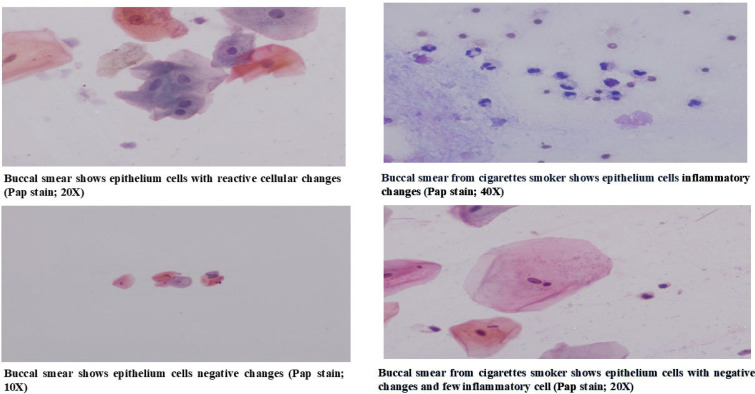
Comparative analysis of cytological changes in the buccal mucosa among traditional cigarette and electronic cigarette users based on cytological findings, September 2023 – June 2024 (N=159)

Buccal smears were collected from the lateral boundary of the buccal cavity using a wooden spatula. The samples were immediately spread onto pre-labeled glass slides and fixed in 95% ethanol for a minimum of 30 minutes. The smears were then stained using the Papanicolaou staining technique (Figure 1), following the protocol described by Salih et al.^12^, with slight modifications. These modifications included rehydration of the smears and an extended staining duration for Harris hematoxylin, increased from 4 to 7 minutes at room temperature. After the staining process, the smears were mounted using Dibutyl Phthalate Xylene (DPX).

### Data analysis

Statistical analysis was conducted using SPSS version 17 (SPSS Inc., Chicago, IL, USA). The Pearson chi-squared test assessed significance, with a p-value threshold of 0.05 at a 95% confidence level.

## RESULTS

The study population had a mean age of 20 years, with an age range of 18–39 years. The distribution of smokers and non-smokers of electronic cigarettes was generally consistent across all age groups. The highest proportion of smokers was observed in the age group of 18–22 years, followed by the age group of 23–39 years. Cytological reactive and inflammatory changes were most frequently observed in the age group of 18–22 years, with 67 out of 146 individuals (45.9%), followed by the 23–39 years age group, with 10 out of 13 individuals (76.9%) (p=0.290).

Cytomorphological analysis using the Papanicolaou (Pap) stain revealed that 82 participants (51.6%) had negative cytological results, 47 participants (29.6%) exhibited reactive changes, and the remaining participants displayed inflammatory changes. Among smokers, 45 individuals (46.4%) demonstrated reactive cytological changes, compared to only 2 non-smokers (3.2%) (p=0.001). Traditional cigarette smokers showed a higher prevalence of reactive cytological changes (18 participants, 51.4%) compared to electronic cigarette smokers (12 participants, 37.5%). Furthermore, dual users of traditional and electronic cigarettes exhibited reactive cellular changes in 15 individuals, accounting for 50.0% of this group.

Reactive cytomorphological changes were predominantly observed in individuals with a smoking history of ≥5 years, accounting for 23 cases (71.8%), compared to 22 cases (33.8%) among those who had smoked for <5 years (p=0.001). Among the smoking population, 47.7% were electronic cigarette users, with >37% demonstrating reactive cytomorphological alterations. A strong association was also evident between the number of cigarettes smoked per day and the occurrence of reactive changes. These alterations were identified in 33.3% of individuals smoking 5–10 cigarettes daily, 57.0% of those smoking >20 cigarettes per day, and 50.0% of dual users (p=0.0001) (Table 1).

**Table 1:** Comparative analysis of cytological changes in the buccal mucosa among traditional cigarette and electronic cigarette users based on smoking duration and status, and number of cigarettes and cytological findings, September 2023–June 2024 (N=159)

*Cytological findings*	*Smoking duration (years)*		*Smoking status*		*Number of cigarettes per day*			
	*<5* *n (%)*	*≥5* *n (%)*	*Non-smokers* *n (%)*	*Dual users* *n (%)*	*5–10* *n (%)*	*11–19* *n (%)*	*≥20* *n (%)*	*E-cigarette daily use* *n (%)*
**Negative**	24 (36.9)	2 (6.3)	56 (90.3)	8 (26.6)	3 (33.3)	3 (25.0)	3 (21.5)	9 (28.1)
**Inflammatory changes**	19 (29.3)	7 (21.9)	4 (6.5)	7 (23.4)	3 (33.3)	2 (16.7)	3 (21.5)	11 (34.4)
**Reactive changes**	22 (33.8)	23 (71.8)	2 (3.2)	15 (50.0)	3 (33.3)	7 (58.3)	8 (57.0)	12 (37.5)
**Total**	65 (100)	32 (100)	62 (100)	30 (100)	9 (99.9)	12 (100)	14 (100)	32 (100)
**Pearson χ^2^**	0.001		0.001					

## DISCUSSION

This study is the first to observe cytopathological changes associated with e-cigarette use in buccal smears of youth in Saudi Arabia. The impact of smoking as a significant risk factor for oral premalignant changes and malignancy is directly associated with the number of cigarettes smoked daily and the duration of smoking. Smoking induces a variety of alterations in the oral mucosa, contributing to a spectrum of diseases ranging from reversible conditions to oral premalignant lesions and malignancies^13^. The type of smoking, such as the use of electronic cigarettes (e-cigarettes), also plays a crucial role. This study aimed to evaluate the effects of electronic cigarettes and traditional cigarette smoke on the buccal mucosa, compared to non-smokers, by assessing cytomorphological changes. Early diagnosis of oral lesions is critical as it greatly influences the success of treatment^14^.

Exfoliative cytology of buccal smears has been widely utilized for assessing epithelial atypical changes and for the early screening and primary diagnosis of premalignant and malignant oral mucosa lesions^7^.

In this study, the smoking duration was categorized into two groups: more than ≥5 years and <5 years. The relationship between the number of cigarettes smoked per day and cytological findings revealed that 33.3% of individuals who smoked 5–10 cigarettes per day exhibited reactive cytological changes. In contrast, 57.0% of individuals who smoked >20 cigarettes per day demonstrated similar reactive changes. These findings align with previous studies that have reported a strong positive association between the occurrence of cytological alterations, such as cytomorphometric and micronuclei changes, and both the frequency and duration of smoking^15-17^. Moreover, the results suggest a possible association between the number of cigarettes consumed per day, regular cigarette smoking, and an increased rate of cytological changes in the buccal mucosa. Aigbogun et al.^18,19^ conducted a study that detected the cytomorphological patterns of buccal smears in passive smokers, active cigarette smokers, and non-smokers. Their findings indicated that cytological cellular changes were more severe in the buccal smears of active cigarette smokers compared to those of passive smokers and non-smokers. The study also determined that cigarette smoking induces DNA impairment and promotes cellular death by enhancing cytological changes in buccal smears, thereby serving as potential indicators for assessing the risk of oral malignancy.

In a cytological comparative study by Kamath et al.^20^ involving smokers and non-smokers, it was demonstrated that cigarette smoking leads to chromosomal damage in the epithelial cells of the buccal mucosa, which is reflected in the increased frequency of micronuclei among smokers. These findings align with the results of our study, which showed that the percentage of reactive cytological changes, indicative of the initial signs of dysplasia, was significantly higher among smokers compared to non-smokers (2–3.2%).

Seifi et al.^10^ evaluated the cytological changes in the buccal mucosa among smokers and waterpipe users, concluding that traditional cigarette smoking had a more pronounced effect on inducing measurable cytometric changes in the buccal mucosa compared to waterpipe use. Our findings corroborate this, as traditional cigarette smokers exhibited a significantly higher prevalence of reactive cytological changes compared to electronic cigarette smokers (51.4% vs 37.5%). Among participants who smoked both traditional and electronic cigarettes, reactive cellular changes were observed in 50.0% of the group. The electronic cigarette, which is becoming increasingly popular, particularly among teenagers and university students, may be comparable to waterpipe smoking in terms of its appeal and usage patterns^21,22^. This trend is especially prevalent in Saudi Arabia, other Arabic countries, and various Asian nations^23^.

Electronic cigarette users often perceive e-cigarettes as less harmful with respect to cytomorphological changes when compared to traditional cigarettes. However, existing literature underscores that both forms of smoking pose significant health risks, including overlapping adverse effects. E-cigarettes deliver active components to the respiratory tract and oral cavity through the aerosolization of a liquid vehicle, which is heated and inhaled. This liquid vehicle typically contains various substances, including possibly tetrahydrocannabinol (THC), flavoring agents, nicotine, and other additives, as well as carrier diluents such as propylene glycol (PG) and vegetable glycerin (VG)^24^.

Although nicotine itself is not classified as a carcinogen, it can be metabolized into nitrosamines^25^, which are well-documented carcinogenic substances. Nitrosamines are present in e-cigarettes and may form during the manufacturing process of the e-liquid or as a result of heating specific ingredients. While existing toxicity data on e-cigarette products primarily focus on the combination of PG and VG^26^, further investigation into the cytological changes and health implications associated with nitrosamines and other harmful substances is urgently needed.

Nitrosamines and other toxic compounds in cigarette smoke have been shown to induce significant cytomorphological changes in buccal mucosa cells^27^. These changes include increased cellular proliferation, nuclear abnormalities, and other precancerous alterations^28^. Although the levels of harmful substances in e-cigarettes are generally lower than those in traditional cigarettes, they still pose a considerable potential risk^29,30^. These levels may contribute to the reactive cytological changes observed in the study group that consumes e-cigarettes.

E-cigarette exposure has been associated with deoxyribonucleic acid damage and oxidative stress-induced cell death. In a mouse model, vaping VG and PG vapors led to epithelial damage^31^. However, studies comparing the toxicological effects of individual constituents in e-cigarette products remain limited. This study hypothesizes that each component of e-cigarettes, including nicotine, could contribute to distinct pathological changes in the respiratory tract and buccal cavity.

### Limitations

This study has several limitations. The sample size may not be large enough to generalize findings to the broader population, as a larger sample would provide more robust statistical power. Additionally, the cross-sectional design restricts the ability to establish causal relationships between cigarette exposure (traditional or electronic) and cytological changes in the buccal mucosa. The study also focuses predominantly on young adults, which may not fully represent cytological changes across different age groups or populations with varying smoking habits. Differences in smoking intensity, duration, and patterns among participants were not accounted for, potentially influencing the observed cytological outcomes. Finally, the collection of buccal smears at a single time point limits the ability to observe temporal or progressive changes in cellular morphology and proliferation.

## CONCLUSIONS

This study demonstrates a significant association between smoking and cytomorphological changes in the buccal mucosa, with higher rates of reactive changes observed in smokers compared to non-smokers. The severity of these changes correlated with smoking duration and intensity, particularly among traditional cigarette and dual smokers. These findings emphasize the cytotoxic effects of smoking on oral epithelial cells and the need for targeted public health strategies to reduce smoking, especially in young adults. Further research is required to explore the long-term impacts of different smoking behaviors on oral health.

## Data Availability

All data generated or analyzed during this study are included in this article. Further inquiries can be directed to the corresponding authors.
